# Towards using bacterial microcompartments as a platform for spatial metabolic engineering in the industrially important and metabolically versatile *Zymomonas mobilis*

**DOI:** 10.3389/fbioe.2024.1344260

**Published:** 2024-01-26

**Authors:** Lior Doron, Dhairya Raval, Cheryl A. Kerfeld

**Affiliations:** ^1^ MSU-DOE Plant Research Laboratory, Michigan State University, East Lansing, MI, United States; ^2^ Department of Engineering, Michigan State University, East Lansing, MI, United States; ^3^ Environmental Genomics and Systems Biology and Molecular Biophysics and Integrative Bioimaging Divisions, Lawrence Berkeley National Laboratory, Berkeley, CA, United States; ^4^ Department of Biochemistry and Molecular Biology Michigan State University, East Lansing, MI, United States

**Keywords:** bacterial microcompartments, *Zymomonas mobilis*, spatial organization, HO shells, synthetic biology

## Abstract

Advances in synthetic biology have enabled the incorporation of novel biochemical pathways for the production of high-value products into industrially important bacterial hosts. However, attempts to redirect metabolic fluxes towards desired products often lead to the buildup of toxic or undesirable intermediates or, more generally, unwanted metabolic cross-talk. The use of shells derived from self-assembling protein-based prokaryotic organelles, referred to as bacterial microcompartments (BMCs), as a scaffold for metabolic enzymes represents a sophisticated approach that can both insulate and integrate the incorporation of challenging metabolic pathways into industrially important bacterial hosts. Here we took a synthetic biology approach and introduced the model shell system derived from the myxobacterium *Haliangium ochraceum* (HO shell) into the industrially relevant organism *Zymomonas mobilis* with the aim of constructing a BMC-based spatial scaffolding platform. SDS-PAGE, transmission electron microscopy, and dynamic light scattering analyses collectively demonstrated the ability to express and purify empty capped and uncapped HO shells from *Z. mobilis*. As a proof of concept to internally load or externally decorate the shell surface with enzyme cargo, we have successfully targeted fluorophores to the surfaces of the BMC shells. Overall, our results provide the foundation for incorporating enzymes and constructing BMCs with synthetic biochemical pathways for the future production of high-value products in *Z. mobilis*.

## 1 Introduction

The use of microorganisms in the production of commercial products is emerging as a valuable strategy because it reduces the production of toxic waste and is considered sustainable, clean, natural, and inexpensive (Adesina et al., 2017). In many cases, the bacterial chassis that is selected to execute the synthetic metabolic function, typically *Escherichia coli* or *Bacillus subtilis*, is chosen because of certain characteristics such as fast growth rate, the ability to survive in a range of different growth conditions, and the availability of genetic modification tools. However, in some cases, these bacterial chassis are not fitted to execute the functions needed for efficient bioproduction (Calero and Nikel, 2018). The integration of synthetic biology and advanced metabolic engineering has enabled the incorporation of non-model organisms as hosts for developing efficient microbial cell factories. These non-traditional organisms typically possess unique enzymatic or fitness advantages, such as increased robustness or efficient metabolism that make them better suited for industrial processes.

*Zymomonas mobilis* (*Z. mobilis)* is an obligatory fermentative alpha-proteobacterium that has attracted significant interest as a platform for the biosynthesis of biofuels due to of its native ability to efficiently metabolize glucose to ethanol rather than to biomass (Xia et al., 2019; Zhang et al., 2019). It possesses several desirable industrial biocatalyst characteristics, such as high productivity, high alcohol tolerance, and a broad pH range for production (pH 3.5–7.5), that make it an ideal platform for industrial-scale production of biofuels and other valuable bioproducts (Yang et al., 2016). Although diverse metabolic engineering approaches have expanded the potential of *Z. mobilis*, for example, by broadening its substrate range to include xylose and arabinose, or by enhancing *Z. mobilis* tolerance to ethanol and lignocellulosic hydrolysate inhibitors (Wang et al., 2018; Zhang et al., 2019), many attempts to redirect metabolic fluxes towards desired products by enhancing native pathways or introducing new heterologous pathways in *Z. mobilis* resulted in the accumulation of toxic or unwanted intermediates (Chen et al., 2013; Yang et al., 2016; Felczak et al., 2019). Therefore, in addition to the linear design of metabolic pathways in *Z. mobilis*, successful engineering must consider the spatial separation of introduced biosynthetic pathway enzymes to cope with challenges such as unproductive or harmful crosstalk (Heinig et al., 2013).

The co-localization of pathway enzymes and their substrates is an attractive approach for multi-enzymatic synthesis in engineered cells. The use of compartmentalization in metabolic engineering has been demonstrated to increase the production efficiency of different metabolites by taking advantage of the endogenous substrate pool in various organelles such as mitochondria (Lv et al., 2016; Sheng et al., 2016), peroxisomes (Gassler et al., 2020), and chloroplasts (Wu et al., 2006). However, with the increasing use of industrial microorganisms to produce high value bioproducts, there is also a requirement to achieve a spatial organization within prokaryotic cells. Bacterial microcompartments (Kerfeld et al., 2018; Sutter et al., 2021) provide a natural model for compartmentalization inside a prokaryotic cell. The utilization of these self-assembling organelles as scaffolds for metabolic enzymes is a sophisticated approach that is becoming widely useful (Kirst and Kerfeld, 2019; Kerfeld and Sutter, 2020; Raba and Kerfeld, 2022). BMC shells natively sequester an enzymatic core that carries out a metabolic pathway. The shell is selectively permeable, functioning as a barrier between the encapsulated enzymes and the cytosol. They have been bioinformatically identified in the majority of bacterial phyla (Sutter et al., 2021) and are known to be involved in CO_2_ fixation in autotrophs (reviewed in Rae et al., 2013; Kerfeld and Melnicki, 2016; Liu, 2022) and in the catabolism of organic substrates such as 1,2-propanediol (Bobik et al., 1999), ethanolamine (Kofoid et al., 1999), small saccharides (Petit et al., 2013; Erbilgin et al., 2014), taurine (Burrichter et al., 2021), and aromatic compounds (Doron et al., 2023). The shell of most BMCs is composed of three types of protein building blocks, which assemble into icosahedral bodies. These include a BMC-H monomer (pfam00936) that assembles into hexamers, BMC-T (2x pfam00936), a pseudohexamer formed from trimers, and BMC-P that assembles into pentamers and cap the vertices (pfam03319). A pore, typically formed at the cyclic symmetry axis of hexamers and pseudohexamers, and can vary in their size or charge, serves as a channel for metabolites to traverse the shell (Kerfeld et al., 2018).

**FIGURE 1:**
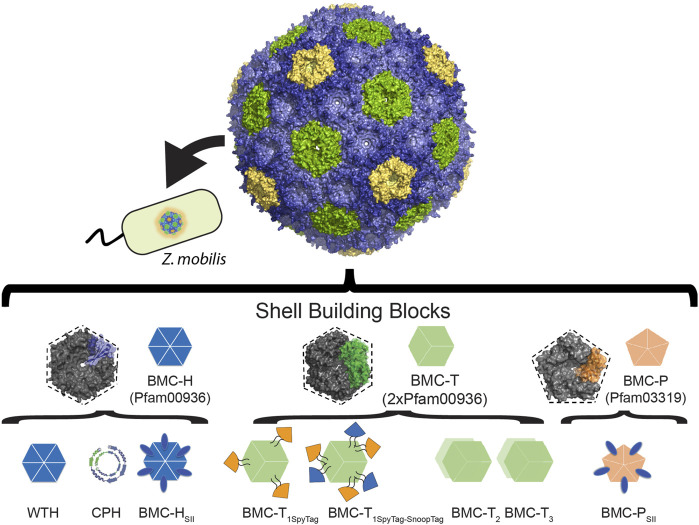
Model of the HO synthetic shell with the functionalized building blocks that were engineered in *E. coli* and applied to *Z. mobilis*. Overview of the modifications of HO shell proteins. SpyTag and SnoopTag split adhesion domains were introduced into a loop within BMC-T_1_ shell proteins to facilitate the encapsulation of cargo proteins. A Strep tag was added to the C-terminus of BMC-P or BMC-H to allow the rapid purification of the loaded shells. Protein cargo can be genetically fused to the C-terminus of WTH (cargo will be displayed on exterior surface of shell) or CPH (cargo will be encapsulated within the lumen of the shell).

The ability to encapsulate many enzymes within a selectively permeable, tunable shell has made the idea of repurposing BMC shells to encapsulate non-native enzymes highly attractive. The development of the synthetic model shell systems derived from the Pdu BMC (Parsons et al., 2010; Nichols et al., 2020), carboxysome (Cai et al., 2016; Sutter et al., 2019; Li et al., 2020), the metabolosome of *Haliangium ochraceum* (HO shells) (Lassila et al., 2014; Sutter et al., 2017), or the GRM BMC (Kalnins et al., 2020), as well as the ability to target non-native cargo into their lumen has paved the way towards designing novel nano bioreactors. Furthermore, in an effort to facilitate the encapsulation efficiency, which was relatively low when native encapsulation methods were used (reviewed in Aussignargues et al., 2015), various synthetic encapsulation methods were developed. One method involves the design of synthetic circularly permuted hexamer (CPH) with an inverted sidedness of its N- and C-terminal residues relative to wild type hexamer (WTH). The direct fusion of protein cargo to WTH from HO (BMC-H) or Pdu (PduA) systems or to their permuted versions, resulted in the displaying of the cargo on the external shell surface or the encapsulation of the cargo within the lumen, respectively (Lee et al., 2018; Ferlez et al., 2019). Another method employs the incorporation of split bacterial adhesion domains, specifically SpyTag (Zakeri et al., 2012) and SnoopTag (Veggiani et al., 2016), into a luminal loop of the HO BMC-T shell protein, and its counterpart domain (SpyCatcher and SnoopCatcher) to a heterologous protein cargo. The interaction between the two domains results in a covalent link between the two proteins and was shown to improve the encapsulation efficiency of various fluorophores and enzymes (Hagen et al., 2018; Kirst et al., 2022). Furthermore, the SnoopTag/SnoopCatcher system does not exhibit cross-reactivity with the SpyTag/SpyCatcher system, making it possible to simultaneously encapsulate multiple enzymes. In addition, fusing protein elements such as affinity tags to the C-terminus of shell proteins, such as BMC-H and BMC-P facilitated the purification of empty or loaded shells (Hagen et al., 2018). Altogether, these features (Figure 1) make the shells of BMCs to be an ideal platform for “bottom-up” approaches to construct synthetic BMCs carrying out entirely novel functions (Kirst and Kerfeld, 2019; Kerfeld and Sutter, 2020). These include the construction of synthetic BMCs that encapsulate pyruvate decarboxylase and an alcohol dehydrogenase from *Z. mobilis* within Pdu shells for the production of ethanol (Lawrence et al., 2014), the encapsulation of HydA, an [FeFe]-hydrogenase and ferrodoxin from the green alga *Chlamydomonas reinhardtii*, within α-carboxysome shells for the production of hydrogen (Li et al., 2020), and the encapsulation of pyruvate formate lyase (PFL) and the acetyl-CoA producing enzyme phosphotransacetylase within HO shells for the production of pyruvate (Kirst et al., 2022).

In this study, we applied tools for constructing and programming shells inside *Z. mobilis*. We show here that the expression of synthetic operons encoding for HO shell proteins in *Z. mobilis* enabled the purification of fully assembled shells and shells that lack pentamers (“uncapped”) at their vertices, which we refer to as wiffle balls. Furthermore, we demonstrated the potential of the *Z. mobilis* shells to encapsulate or display fluorophores as cargo. These results provide the proof-of-concept required to show that the BMC shell system can be a sophisticated compartmentalization strategy for improving bioproduct synthesis in the industrially relevant microorganism *Z. mobilis*.

## 2 Materials and methods

### 2.1 Plasmid construction of BMC operon, superfolderGFP-fused hexamers, and SpyCatcher-tagged fluorophores

Primers and plasmids used in this study are listed in Supplementary Table S1.

The *Z. mobilis* codon-optimized minimal HO shell operon encodes BMC-H, BMC-T_1SpyTag_, and a Strep-tagged BMC-P (P_SII_) from *H. ochraceum*. The operon was synthesized with NdeI and BamHI restrictions sites and cloned into an isopropyl β-D-1-thiogalactopyranoside (IPTG)-inducible expression plasmid pRL814 expression plasmid (Ghosh et al., 2019). The minimal and full HO wiffle ball operons were PCR-amplified from plasmids used (Kirst et al., 2022) and cloned into pRL814 using Gibson assembly (Gibson et al., 2009). *Z. mobilis*-compatible superfolderGFP (sfGFP) with an optimized ribosome binding site (Lal et al., 2019; Lal et al., 2021), and mScarlet (Hall et al., 2023) fluorophores were provided by the lab of Patricia Kiley and Robert Landick at the University of Wisconsin-Madison, respectively. The WTH-sfGFP and CPH-sfGFP genes were cloned into an IPTG-inducible expression plasmid pIND4 (Ind et al., 2009) using Gibson assembly. Specifically, the gene for WTH was PCR amplified from the pRL814 codon-optimized minimal HO shell operon while the CPH gene was PCR-amplified from a plasmid used (Ferlez et al., 2019). The SpyCatcher domain, which was PCR-amplified using a plasmid from Hagen et al. (2018), was fused to sfGFP or mScarlet-I using Gibson assembly. The gene for sc_mScarlet was cloned into a tetracycline controlled TetR-Ptet plasmid (pTet) pYL48 (Liu et al., 2020) using Gibson assembly. pYL48 was provided as a gift from the lab of Robert Landick at the University of Wisconsin-Madison.

### 2.2 Growth of bacterial strains

A *Zymomonas mobilis* quadruple mutant strain (Δ*hsdS*_*c*_ Δ*hsdS*_*p*_ Δ*mrr* Δ*cas3*), also known as PK15509 (Lal et al., 2021), was cultured in partially aerobic conditions with stirring in a sealed 2L bottle in rich medium ZRMG (1% yeast extract, 0.2% KH_2_PO_4_, 2% glucose). Induction was carried out by adding 0.5 mM IPTG or 0.5 mM IPTG+200 ng/mL anhydrous tetracycline (aTc) at mid-exponential phase (OD600 nm = 0.3–0.4) and continued to grow with cap partially open with stirring for 4 h at 30°C. Spectinomycin, Kanamycin, and Chloramphenicol were used for bacterial selection at a final concentration of 100, 150, and 50 μg/mL, respectively.

### 2.3 Conjugation

*E. coli* donor strain WM6026, an m-diaminopimelate (DAP) auxotrophic strain (Blodgett et al., 2007), was used to conjugate plasmids into the recipient strain *Z. mobilis* ZM4 (PK15509). WM6026 was grown aerobically for ∼16 h at 37°C in LB containing DAP (0.1 mM) with appropriate antibiotic. The overnight culture was then subcultured into LB medium lacking antibiotic to an OD_600_ of 0.15 and grown to an OD_600_ of 0.5. *Z. mobilis* was incubated in 5 mL of ZRMG without shaking at 30°C until a late exponential phase. For conjugation, 1 mL of cells, adjusted to OD_600_ of 0.5, of both recipient and donor washed twice (4,000 × g for 5 min) and gently resuspended in an equal volume of fresh medium without antibiotic or DAP. The cells were combined in a 2 mL microfuge tube and briefly centrifuged at 17,300 × g for 30 s, and supernatant was decanted. The pellet was resuspended in the remaining medium and the *Z. mobilis* and *E. coli* mixed solution was placed as drops on a prewarmed (30°C–37°C) ZRMG containing 1% tryptone, 0.15 mM DAP agar plate. Plates were incubated overnight at 30°C (12–15 h) and drops were collected from plates and resuspended in 1 mL ZRMG liquid media. The conjugation mixture was then vortexed for 10 s, spun at 17,300 × g, and the pellet was resuspended in 1 mL ZRMG liquid medium. The suspensions were incubated without shaking at 30°C for 3 h, and 100 μL of the undiluted, 10-fold, and 100-fold diluted cell suspension were plated on ZRMG agar plates containing the appropriate antibiotics and incubated at 30°C for 4 days until the appearance of colonies.

### 2.4 Purification of empty or loaded shells from *Z. mobilis*

A cell pellet of *Z. mobilis* strains expressing HO minimal shells, minimal wiffle balls, or full wiffle balls with or without cargo was resuspended in tris-buffered saline (50 mM Tris, 200 mM NaCl, pH 8.0; TBS 50/200 hereafter), and lysed by French press at 2 × 1,100 psi in the presence of SigmaFast protease inhibitor, 0.1 mg/mL lysozyme, and 1 mg/mL DNase (Sigma Aldrich, St. Louis United States). The lysate was further clarified by centrifugation at 30,000 ×g for 30 min and supernatant was loaded on 30% sucrose cushion and centrifuged in a Beckman type 70 Ti (fixed-angle) rotor for 16 h at 181,000 ×g at 4°C to size-differentiate unincorporated shell proteins and cargo before application onto the StrepTrap column. Shells were eluted off the column using TBS 50/200 + 2.5 mM desthiobiotin. Shell-containing elution fractions were pooled and concentrated in a 15 mL 100 kDa MWCO filter (Amicon) and stored in 4°C after the addition of 0.02% sodium azide as a preservative.

### 2.5 SDS-PAGE of protein preparations

Purified shell preparations were typically normalized to A280 = 1, boiled and denatured in reducing sample buffer and loaded on 18% polyacrylamide gels. Gels were washed and stained with Coomassie blue and imaged with ChemiDoc™ XRS + System. For imaging the GFP or mScarlet-I intrinsic fluorescence signal, protein samples were heated at 65°C for 15 min in reducing sample buffer and loaded on 18% polyacrylamide gels. Gels were washed and imaged with ChemiDoc™ XRS + System equipped with a bandpass filter for GFP detection (520 nm) and mScarlet-I detection (595 nm). After detection of the fluorescent bands, the gels were stained with Coomassie Blue to detect total protein.

### 2.6 Dynamic light scattering analysis

Dynamic light scattering data were collected using a Wyatt DynaPro Nanostar. 10 μL of the concentrated StrepTrap elution fractions were loaded into a 10 μL (1 × 1 × 10 mm) cuvette and three scans, each consisting of 20 5-s acquisitions, were used to measure the size distribution of HO shells and wiffle balls in solution. Shell diameter was averaged from three technical repeats.

### 2.7 Transmission electron microscopy analysis

Purified shells were imaged by negative stained TEM on a JEOL 100CXII microscope operated at an accelerating voltage of 100 kV using a Gatan Orius SC200 CCD camera. Purified shells were diluted 10-fold in HPLC-grade water and 5 μL of each sample was applied to 150 mesh carbon-coated copper grids (Electron Microscopy Sciences, Hatfield United States) for 30 s, wicked dry, stained for 15 s with 1% uranyl acetate, and again wicked dry before imaging.

### 2.8 Fluorescence microscopy

For microscopy images, 1 mL of IPTG-induced cells were pelleted by centrifugation at 5,000 ×g for 5 min and the pellet was resuspended in 100 μL of sterile H_2_O. A 5 μL aliquot was transferred to a 3% (w/v) agarose pad. The pad was placed onto a #1.5 glass coverslip for imaging. Fluorescence images were taken with a Zeiss Axio Observer D1 microscope (63 × 1.3 NA oil lens) with an Axiocam 503 (mono-chrome) camera using light from X-Cite 120Q (Lumen Dynamics, Mississauga, Canada). For fluorescent sc_sfGFP signals, we used filter set 46 (000000-1196-681): excitation BP 500/20, emission BP 535/30, and beam splitter FT515. For mScarlet-I we used excitation BP 590/20, emission BP 635/30.

## 3 Results

### 3.1 Design of HO shell and wiffle ball synthetic operons

**FIGURE 2:**
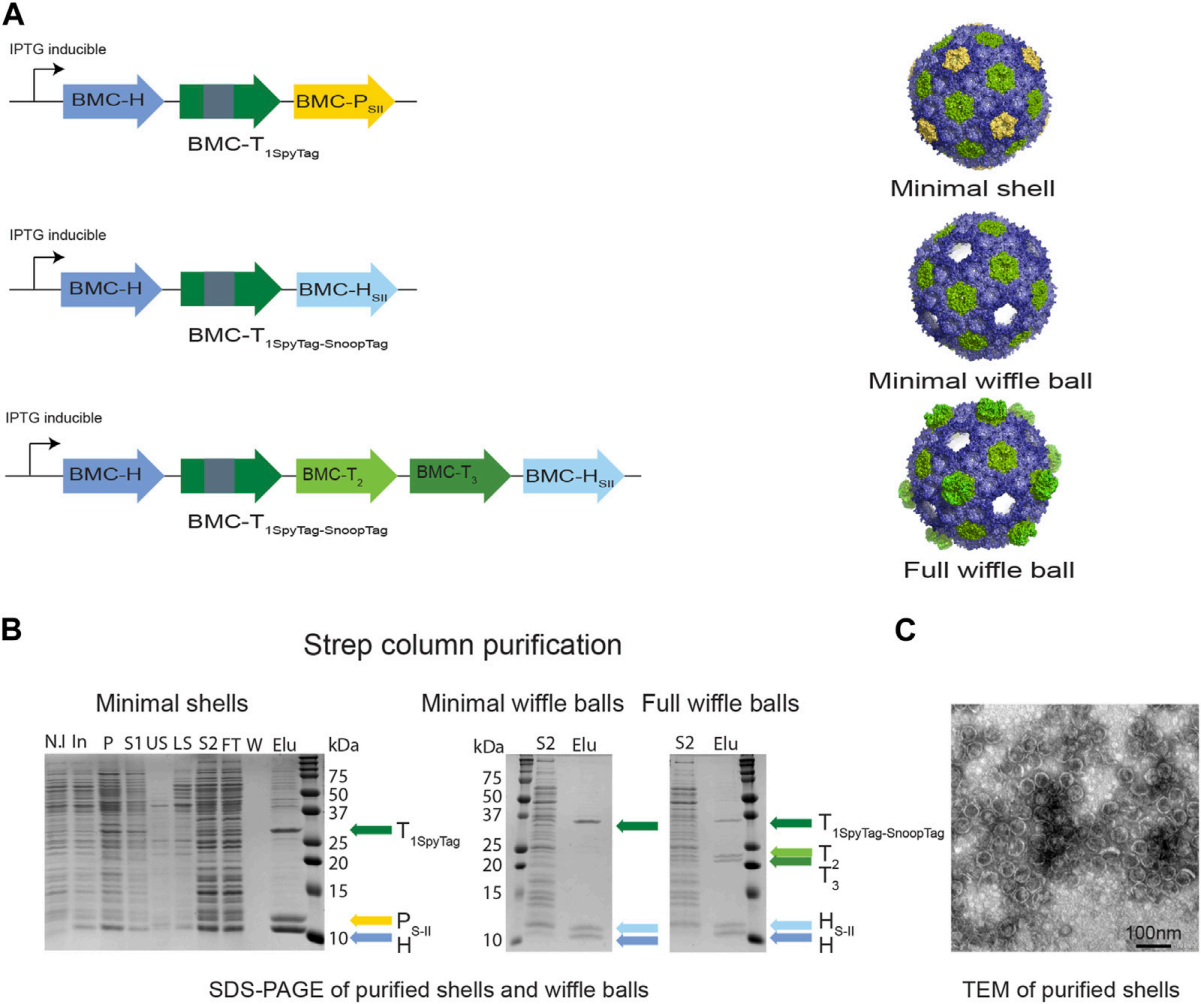
Purifying intact empty BMC shells and wiffle balls from *Z. mobilis*. **(A)** Diagram of synthetic HO operons and the resulting shell architectures. All synthetic operons encode shell proteins under the control of a T7 IPTG-inducible promotor. A strep tag was added to either BMC-P (minimal shells) or to BMC-H (minimal and full wiffle balls). **(B)** Expression and purification of intact empty shells and wiffle balls in *Z. mobilis.* Assembly of shells and wiffle balls were confirmed by SDS-PAGE stained with Coomassie Blue **(B)** and transmission electron microscopy analysis **(C)** of purified shells. TEM analyses revealed the presence of shells (diameter of 39 nm) on a grid negatively stained with uranyl acetate. Colored arrows mark the different shell proteins. SDS-PAGE key: N.I- non induced, In- induced cells, P- insoluble fraction, S1- soluble clear lysate, US- upper sucrose cushion fraction, LS- lower sucrose cushion fraction, S2- resuspended bottom pellet, FT- StrepTrap flow-through fraction, W- wash fraction, Elu-elution fractions.

A synthetic full HO shell is encoded by five genes: BMC-H, three BMC-T genes (T_1_, T_2_, T_3_), and one BMC-P gene (Lassila et al., 2014; Sutter et al., 2017). To introduce the HO shell into *Z. mobilis*, we designed a simplified *Z. mobilis* codon-optimized synthetic operon that includes the minimal (i.e., minimal shell) set of genes (BMC-H, BMC-T_1_, BMC-P) that were shown to be required to form 40 nm diameter icosahedral shells in *E. coli* (Hagen et al., 2018). This operon was synthesized by the Joint Genome Institute as part of a collaboration with the Great Lake Bioenergy Research Center. Additionally, we used non-codon optimized synthetic operons that were designed in a previous study (Kirst et al., 2022) and shown to produce minimal (BMC-H and BMC-T_1_) or full wiffle balls (BMC-H, BMC-T_1_, BMC-T_2_, and BMC-T_3_). The wiffle balls lack pentamers in their vertices to allowing the exchange of large molecules/proteins across the shells. To enable efficient encapsulation of cargo proteins, we used two versions of a modified BMC-T_1_. The first version, which we included in our codon-optimized minimal shell operon includes only the SpyTag bacterial adhesion domain in its internal loop (T_1Spy_) (Hagen et al., 2018), while the second version, which is encoded in the wiffle ball operons, includes both the SpyTag and SnoopTag adhesion domains in its internal loop (T_1Spy-Snoop_) to allow the encapsulation of multiple proteins (Kirst et al., 2022). Additionally, we used Strep-II-tagged BMC-P (P_SII_) as a strategy to facilitate purification of empty or loaded shells using complementation-based affinity purification (CAP) (Hagen et al., 2018), or BMC-H (H_SII_) to purify wiffle balls (Figure 1). To express the three synthetic operons in *Z. mobilis* we cloned them into the *Z. mobilis* expression vector pRL814 (Ghosh et al., 2019) downstream to an IPTG-inducible promoter. Overall, we constructed three pRL814 expression plasmids (Figure 2A) encoding for HO BMC-HT_1spy_P_SII_ (minimal shells), HO BMC-HT_1Spy-Snoop_H_SII_ (minimal wiffle balls), and HO BMC-HT_1Spy-Snoop_T_2_T_3_H_SII_ (full wiffle balls).

### 3.2 Purification of HO shells and wiffle balls from *Z. mobilis*

To verify the assembly of shells in *Z. mobilis* we aerobically grew the *Z. mobilis* codon-optimized minimal shell strain in rich media and attempted to purify empty minimal HO shells from the IPTG-induced cells using the CAP technique. Analysis of a concentrated eluate from a StrepTrap affinity column by SDS-PAGE reveals that highly pure shell particles can be obtained via affinity purification of the Strep-tagged pentamer (Figure 2B left panel). The presence of both BMC-H and BMC-T_1Spy_ in the elution fraction together with BMC-P_SII_ demonstrates the ability of BMC-P_SII_ to pull down the other shell components and suggests formation and assembly of intact minimal shells *in vivo*. To confirm the assembly of HO shells, we analyzed the StrepTrap concentrated eluate using dynamic light scattering (DLS) and transmission electron microscopy (TEM). Analysis of the eluate by DLS indicate the presence of particles with a diameter of ∼39 nm (Supplementary Figure S1), while negatively-stained EM grids revealed the presence of morphologically homogeneous intact shells, with an average diameter of 39 nm, similar to the morphology and size of shells obtained in previous studies (Lassila et al., 2014; Sutter et al., 2017; Hagen et al., 2018; Ferlez et al., 2019) (Figure 2C).

We next attempted to express and purify the BMC-P-lacking minimal and full wiffle balls from *Z. mobilis*. We were able to detect the presence of protein bands corresponding to wild type BMC-H, BMC-H_SII_ and the BMC-T_1Spy-Snoop_ in both StrepTrap concentrated eluates on SDS-PAGE (Figure 2B middle panel), as well as two additional protein bands corresponding to the BMC-T_2_ and T_3_ only in the eluate of the full wiffle balls (Figure 2B right panel). These results demonstrate that the Strep-tagged BMC-H was able to pull down the other shell proteins, as with the Strep-tagged pentamer in the minimal shell strain, and exemplify our ability to isolate shells and wiffle balls from total crude extract in *Z. mobilis*.

### 3.3 Targeting cargo fluorophores to the interior and exterior surfaces of HO shells and wiffle balls in *Z. mobilis* using various scaffolding strategies

**FIGURE 3:**
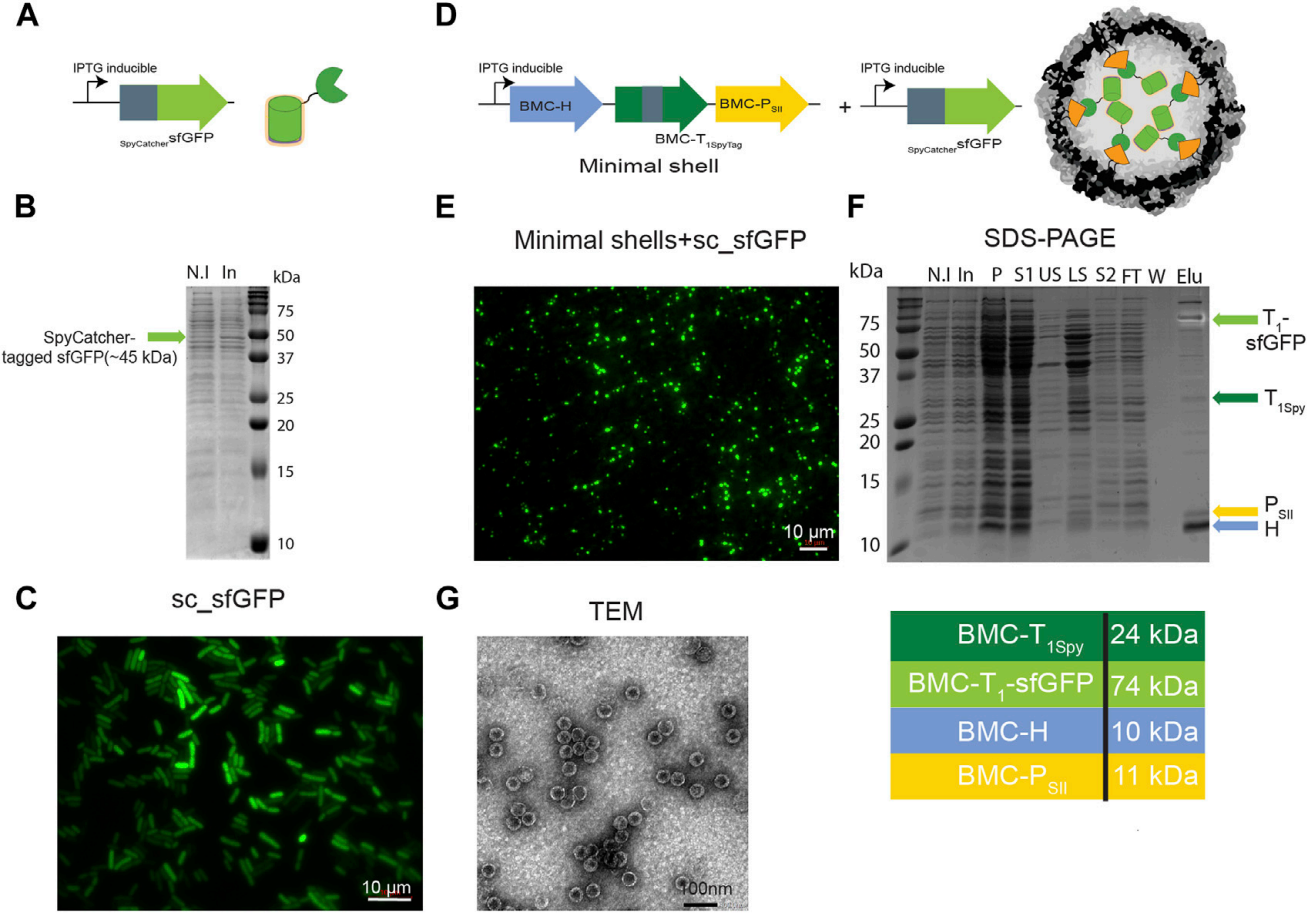
Targeting cargo to minimal shells in *Z. mobilis* using SpyCatcher-tagged GFP. Left panel: Analysis of *Z. mobilis* cells expressing pIND4 sc_sfGFP. *Z. mobilis* cells transformed with pIND4 sc_sfGFP were induced with IPTG and analyzed by fluorescence microscopy and SDS-PAGE. **(A)** Diagram of SpyCatcher-tagged sfGFP gene and the resulting architecture. SpyCatcher domain (depicted by gray rectangle) was fused to the N-terminus of sfGFP gene (depicted by green arrow) downstream to an IPTG-inducible promoter. **(B)** Analysis of *Z. mobilis* cells expressing pIND4 sc_sfGFP by SDS-PAGE. Expression of sc_sfGFP was verified by comparing non-induce (N.I) with induced (In) cells. **(C)** Analysis of *Z. mobilis* IPTG-induced cells expressing pIND4 sc_sfGFP under fluorescent microscope. Right panel: Analysis of *Z. mobilis* cells expressing pIND4 sc_sfGFP and pRL814 minimal shell operon. *Z. mobilis* cells co-transformed with pIND4 sc_sfGFP and pRL814 minimal shell operon plasmids were induced with IPTG and the targeting of sc_sfGFP into the shells was analyzed by fluorescent microscopy, SDS-PAGE, and TEM analyses of the purified shells from a StrepTrap affinity column. **(D)** Diagram of a minimal shell operon and SpyCatcher-tagged sfGFP gene and their resulting architecture. **(E)** Analysis of *Z. mobilis* IPTG-induced cells under a fluorescent microscope. **(F)** Analysis of the purified shell proteins on SDS-PAGE and their expected sizes in kDa. Shell proteins are marked with colored arrows. Detection of the T_1_-sfGFP conjugates (light green arrow) was performed by exposing the gel to UV light prior to its staining with Coomassie Blue. SDS-PAGE key: N.I- non induced, In- induced cells, P- insoluble fraction, S1- soluble clear lysate, US- upper sucrose cushion fraction, LS- lower sucrose cushion fraction, S2- resuspended bottom pellet, FT- StrepTrap flow-through fraction, W- wash fraction, Elu-elution fractions. **(G)** Analysis of the purified assembled sfGFP-loaded shells using TEM.

As a proof-of-concept for the potential of HO shells to be loaded or surface decorated with cargo in *Z. mobilis*, we either genetically fused the SpyCatcher domain to the N-terminus of superfolder-GFP (sc_sfGFP) or mScarlet-I (sc_mScarlet) or genetically fused sfGFP directly to the C-terminus of wild type BMC-H (WTH-GFP) or circularly permuted BMC-H (CPH-GFP). Previous studies have established that the termini of wildtype HO BMC-H hexamers are surface exposed (Sutter et al., 2017; Ferlez et al., 2019); indeed, this appears to be a general feature of BMC shell hexamers (Sutter et al., 2017; Sutter et al., 2019; Kalnins et al., 2020). In contrast, circular permutation of secondary structure elements in the HO BMC-H has demonstrated that this orients the C-terminus to the interiors. These studies confirmed structural observations of the orientation of WTH and CPH-fused fluorophores as external and luminal-targeted cargo, respectively, using a proteolysis analysis (Ferlez et al., 2019). FRET analysis, likewise, confirmed the luminal orientation of the SpyCatcher-fused fluorophore (Kirst et al., 2022). The genes for sc_sfGFP, WTH-GFP, and CPH-GFP were cloned separately into the pIND4 plasmid, while the gene for sc_mScarlet was cloned into the pYL48 plasmid, and expressed in *Z. mobilis*. We first analyzed whether SpyCatcher-tagged cargo could be loaded to HO shells in *Z. mobilis* (Figure 3). For that reason, we either co-transformed pIND4 sc_sfGFP and pRL814 minimal shell expression plasmids (Figure 3D) or transformed pIND4 sc_sfGFP on its own (Figure 3A) and examined the IPTG-induced cells under a fluorescence microscope. In contrast to the induction of the sc_sfGFP on its own (Figure 3B), which resulted in diffused fluorescent signal (Figure 3C), the co-induction of the minimal shells and sc_sfGFP resulted in the formation of small fluorescent circular bodies that were located mostly at a pole of the cells (Figure 3E). This could be the result of the encapsulation of sc_sfGFP within shells that aggregated or the aggregation of only T_1SpyTag∼SpyCatcher_sfGFP conjugates. To verify that indeed sfGFP was loaded into HO minimal shells, we isolated the shells using the CAP method via the BMC-P_SII_ and analyzed the purified proteins on SDS-PAGE (Figure 3F). In addition to the shell proteins that constitute the minimal shells, we identified a fourth ∼75 kDa protein band (Figure 3F light green arrow). Analysis of the gel under UV light to detect the intrinsic sfGFP fluorescence confirmed the ∼75 kDa protein band as the T_1_-sfGFP conjugate. Furthermore, we also noted that the non-conjugated BMC-T_1Spy_ protein band almost completely disappeared in shells purified from cells coexpressing minimal shells and sc_sfGFP (Figure 3F, dark green arrow), compared to the relative abundance of BMC-T_1Spy_ when only minimal shells were being expressed (Figure 2B, dark green arrow). This is most likely due to the interaction of the non-conjugated BMC-T_1Spy_ with sc_sfGFP which lead to the formation of T_1SpyTag∼SpyCatcher_sfGFP conjugates. Finally, TEM analysis of the concentrated elution sample on a negatively stained grid confirms the presence of shells with a diameter of ∼42 nm (Figure 3G).

**FIGURE 4:**
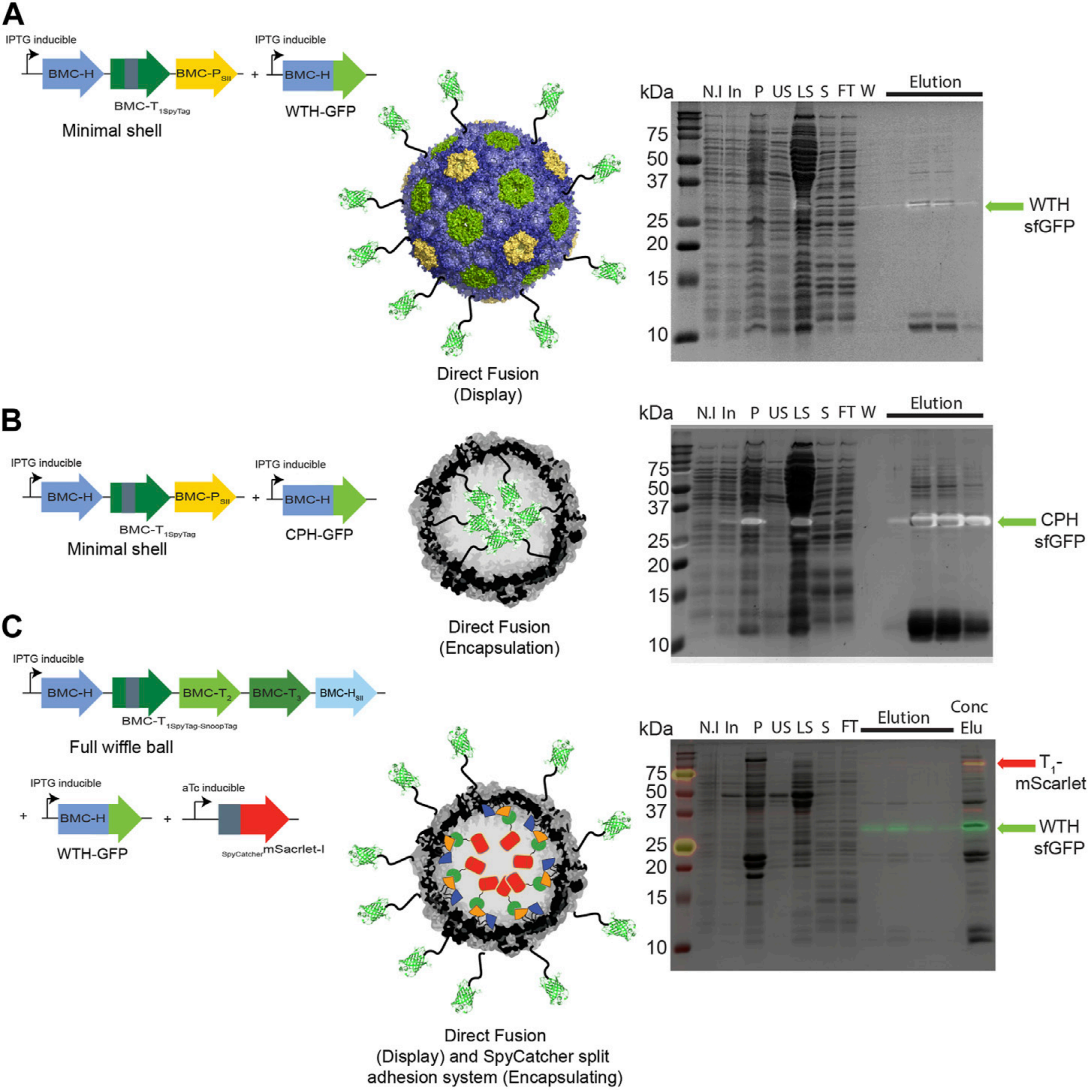
Targeting cargo to minimal shells and full wiffle balls in *Z. mobilis* using different scaffolding strategies. Shell proteins from IPTG-induced cells expressing pRL814 minimal HO shells operon and pIND4 WTH-sfGFP **(A)**, or CPH-sfGFP **(B)**, or from IPTG and aTc-induced cells expressing the pRL814 full HO wiffle ball operon, pIND4 WTH-sfGFP, and pTet sc_mScarlet-I **(C)**, were purified on StrepTrap affinity column and analyzed by SDS-PAGE. Left panel: Diagram of operons and genes used in the analysis and their resulting architecture. Right panel: SDS-PAGE analyses of purified HO shells and wiffle ball proteins from StrepTrap affinity column purifications. Detection of the sfGFP-fused hexamers (light green arrow) or T_1_-mScarlet-I conjugates (red arrow) was performed by exposing the gels to UV light prior to their staining with Coomassie Blue. SDS-PAGE key: N.I- non induced, In- induced cells, P- insoluble fraction, US- upper sucrose cushion fraction, LS- lower sucrose cushion fraction, S- resuspended bottom pellet, FT- StrepTrap flow-through fraction, W- wash fraction, Elu-elution fractions, Conc Elu-pooled and concentrated elution fraction using a 15 mL 100 kDa MWCO filter (Amicon).

We then investigated whether a direct fusion scaffolding strategy could be used to target cargo to the interior and exterior surfaces of HO shells. We co-expressed WTH-GFP (Figure 4A) or CPH-GFP (Figure 4B) with minimal HO shells and pulled down the shell proteins from crude cell extracts. As with the SpyCatcher-tagged GFP, concentrated eluates of both purifications revealed an additional fourth protein band in the size of ∼35 kDa that had an intrinsic sfGFP fluorescence signal on SDS-PAGE, which corresponds to either WTH-GFP (Figure 4A right panel) or CPH-GFP (Figure 4B right panel). The detection of assembled shells on TEM grids (Supplementary Figures S2A, B) substantiate the successful assembly of the purified GFP-loaded or decorated HO shells in *Z. mobilis*. We additionally tested the ability to simultaneously encapsulate and decorate the external surface of wiffle balls with cargo proteins in *Z. mobilis* by co-expressing full wiffle balls, WTH-GFP, and sc_mScarlet. Analysis of the purified proteins from the IPTG and aTc-induced cells on SDS-PAGE revealed the presence of two additional bands with an intrinsic sfGFP or mScarlet fluorescence signal at the size of ∼35 kDa and ∼70 kDa, corresponding to the WTH-GFP and T_1_-mScarlet conjugate, respectively (Figure 4C right panel). TEM analysis of the concentrated elution sample of WTH-GFP-mScarlet-targeted wiffle balls confirmed the presence of assembled shells with an average diameter of 39 nm (Supplementary Figure S2C).

## 4 Discussion

The potential for encapsulating synthetic biochemical pathways into BMC shells in industrially important bacterial hosts has many applications in metabolic engineering (Kerfeld and Sutter, 2020; Stewart et al., 2021; Liu, 2022; Abrahamson et al., 2023). It is especially relevant to pathways with poor enzyme kinetics, or prone to crosstalk with endogenous host pathways or those that generate volatile or toxic intermediates that might disrupt the metabolism of the cell. Although the encapsulation of metabolic enzymes in BMC shells in non-native organisms has mostly been confined to *E. coli* including the construction of shell-based nano bioreactors for production of ethanol (Lawrence et al., 2014), 1,2-propanediol (Lee et al., 2016), hydrogen (Li et al., 2020), and pyruvate (Kirst et al., 2022), several recent studies have introduced BMC gene clusters or synthetic operons encoding BMC shell proteins also into various industrial bacterial hosts. These include the established industrial workhorse for the production of amino acids, *Corynebacterium glutamicum* (Baumgart et al., 2017; Huber et al., 2017), the Gram-positive model organism *Bacillus subtilis* (Wade et al., 2019), and several *Pseudomonas* species (Graf et al., 2018). These studies demonstrated the assembly of shells or BMCs of either the Pdu system from *Citrobacter freundii* (Huber et al., 2017), *Salmonella enterica* (Graf et al., 2018), and the thermophile *Parageobacillus thermoglucosidasius* (Wade et al., 2019), or the α-carboxysome shell from *Halothiobacillus neapolitanus* (Baumgart et al., 2017).

In this study, we took a synthetic biology approach and introduced the HO shell system into *Z. mobilis*. Our ultimate objective is to establish BMC-based spatial organization, with the long-term aim of minimizing the loss of intermediate metabolites and enhancing pathway flux in *Z. mobilis*. This can be done by localizing metabolic enzymes that catalyze sequential steps on the HO BMC shell and wiffle ball scaffolds. To utilize the HO shells and wiffle balls in *Z. mobilis*, it was first necessary to demonstrate the ability to express the different shell proteins in *Z. mobilis* and to subsequently purify the assembled shells using the CAP method (Hagen et al., 2018). Our ability to identify the different shell components on SDS-PAGE following their purification on a StrepTrap affinity column, after adding a sucrose cushion step to get rid of unbound shell proteins (Figure 2B), as well as to visualize intact assembled shells on negatively stained grids (Figure 2C), confirm the successful assembly of HO shells in *Z. mobilis*. Furthermore, our results demonstrate the advantage of using the CAP method in purifying shells in a rapid and simple way from crude Zymomonas cell extract and offer an advantage over other shell systems that require tedious and long purification processes. By adding affinity tags to the C-terminus of HO-BMC-P or BMC-H, shells and wiffle balls can be efficiently purified and bioproducts that were produced within the shells can be rapidly extracted. This is especially valuable for bioproducts that are prone to degradation or oxidation.

In addition to our ability to purify assembled shells or wiffle balls, it was also essential to validate the functionality of various scaffolding strategies that were developed for the HO shell system in *E. coli* over the years for targeting cargo proteins to the interior and exterior parts of the shells. These include the direct fusion of cargo to WTH or CPH proteins and the exploitation of the SpyTag-SpyCatcher/SnoopTag-SnoopCatcher split adhesion bacterial systems that were elegantly and uniquely incorporated into HO shells and wiffle balls. The HO shells are structurally characterized (Sutter et al., 2017) showing that the N and C-termini of the HO hexamer are surface exposed. Additionally, previous studies confirmed the orientation of WTH and CPH-fused fluorophores as external and luminal-targeted cargo, respectively, using a proteolysis analysis (Ferlez et al., 2019), and the orientation of the SpyCatcher-fused fluorophore as luminal using FRET analysis (Kirst et al., 2022). The formation of small circular bodies in *Z. mobilis* cells expressing sc_sfGFP and shell proteins (Figure 3E) and the identification of additional protein bands with intrinsic sfGFP or mScarlet fluorescence signal on SDS-PAGE (Figures 3F, 4 right panels) following purification from StrepTrap affinity column, demonstrate the targeting of fluorophores to shells. These findings serve as a proof-of-concept for our ability to assemble and purify shells with cargo on the exterior and interior surfaces of the shell in *Z. mobilis*. Furthermore, our ability to simultaneously encapsulate and decorate the external surface of the shells and wiffle balls with proteins of choice shows that the external surface of a shell can be functionalized with an enzyme that could potentially create a high local concentration of substrate proximal to the shell, which would enhance the enzymatic reactions of the encapsulated enzymes via increased diffusion of substrates into the shell. By achieving BMC shell-based compartmentalization in *Z. mobilis* and validating the functionality of the different scaffolding strategies, we are providing evidence for the feasibility of future design and construction of synthetic shell-insulated metabolic pathways of up to four enzymes (three encapsulated and one displayed) in *Z. mobilis*. This will further advance the use of biocatalyst unique characteristics that *Z. mobilis* has to offer for the industrial-scale production of biofuels and other valuable bioproducts. It is still debatable whether the uncapped shells should be defined as a separate compartment. At the very least, wiffle balls can be considered three-dimensional scaffolds for the immobilization and co-localization of cargo. In terms of a defined compartment, on the one hand, the absence of the pentamer in the shell vertices leaves a 5 nm hole that allows the crossing of oxygen, enzymes, or other metabolites into the shells. On the other hand, the encapsulation of the metabolic enzymes in close proximity to each other ensures a quick conversion of the intermediates and prevents their diffusion to competing off-branching pathways in the cytosol. This facilitates the continuation of the pathway and increases final product yield. Theoretically, an isolated wiffle ball can be constructed if the regeneration of co-factors occurs within the compartment by the encapsulated enzymes. This was demonstrated with the activity of the sFUT module, where the acetyl-CoA, which is condensed with formate to make pyruvate by PFL activity, is regenerated by the activity of the encapsulated phosphotransacetylase (Kirst et al., 2022).

## Data Availability

The original contributions presented in the study are included in the article/Supplementary Material, further inquiries can be directed to the corresponding author.

## Supplementary material

SUPPLEMENTARY FIGURE S1 Structural characterization of StrepTrap-purified *in vivo* assembled minimal HO shells, minimal HO wiffle balls, and full HO wiffle balls. Dynamic light scattering analysis (left panel) and TEM analysis (right panel) of minimal HO shells **(A)**, minimal HO wiffle balls **(B)**, and full HO wiffle balls **(C)**. The hydrodynamic diameter of the shells is labeled on the plot. The x-axis is logarithmically spaced.

SUPPLEMENTARY FIGURE S2 Characterization of shells and wiffle balls loaded or decorated with fluorophores. Shell proteins from IPTG-induced cells expressing the pRL814 minimal HO shell operon and pIND4 WTH-sfGFP **(A)**, or pIND4 CPH-sfGFP **(B)**, or from IPTG and aTc-induced cells expressing pRL814 full HO wiffle balls, pIND4 WTH-sfGFP, and pTet sc_mScarlet-I **(C)** were purified off a StrepTrap affinity column and analyzed on TEM. Left panel: Diagram of operons and genes used in the analysis. Right panel: Analysis of the purified assembled loaded shells **(A,B)** and wiffle balls **(C)** using TEM.

SUPPLEMENTARY TABLE S1 Plasmids and primers used in this study.
