# Supplementary figures and images for: Towards using bacterial microcompartments as a platform for spatial metabolic engineering in the industrially important and metabolically versatile Zymomonas mobilis
Source: Front Bioeng Biotechnol. 2024 Jan 26;12:1344260. doi: 10.3389/fbioe.2024.1344260 (PMC10853475; doi:10.3389/fbioe.2024.1344260)

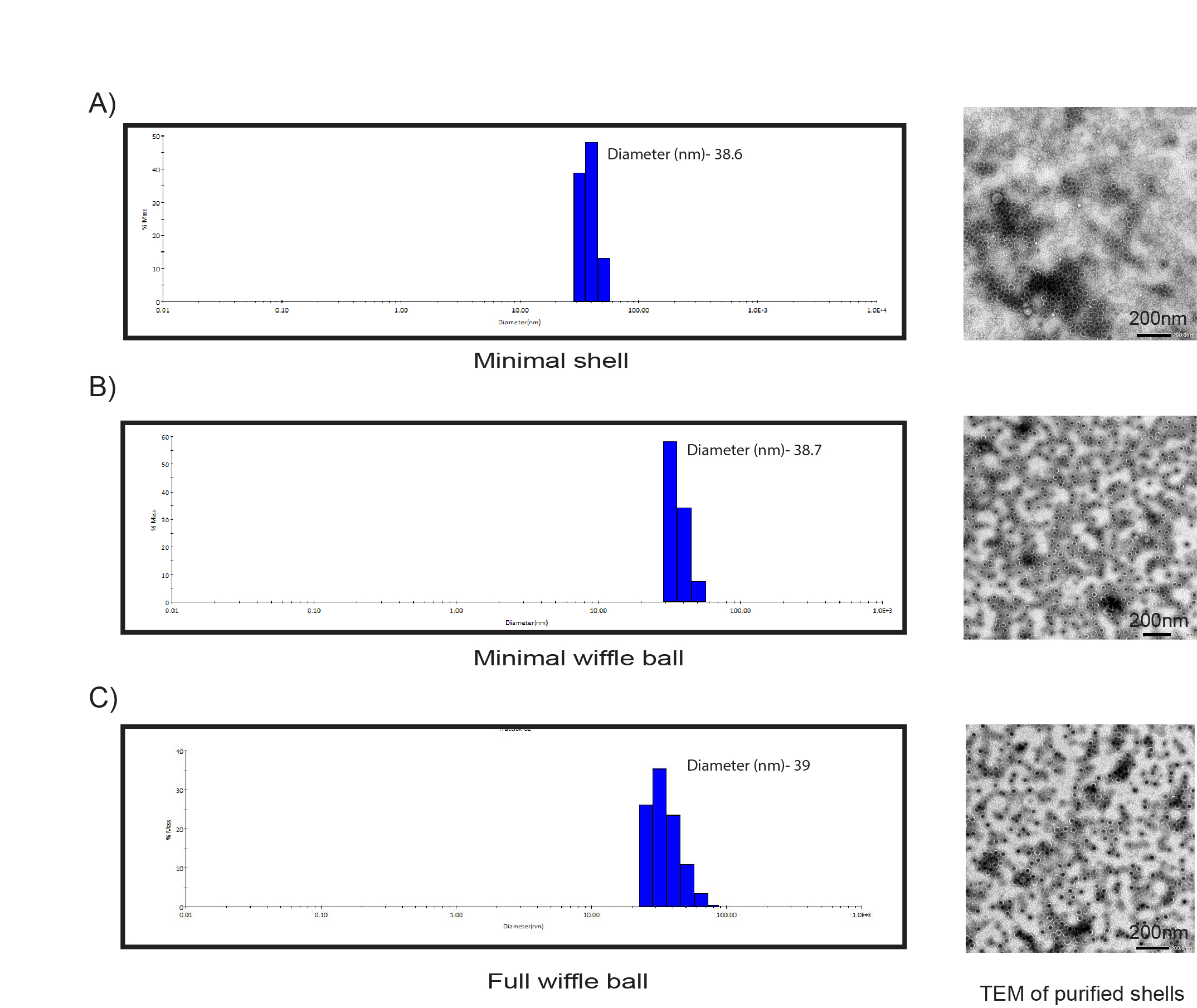

Supplement: Supplementary file 1 [file Image1.JPEG]

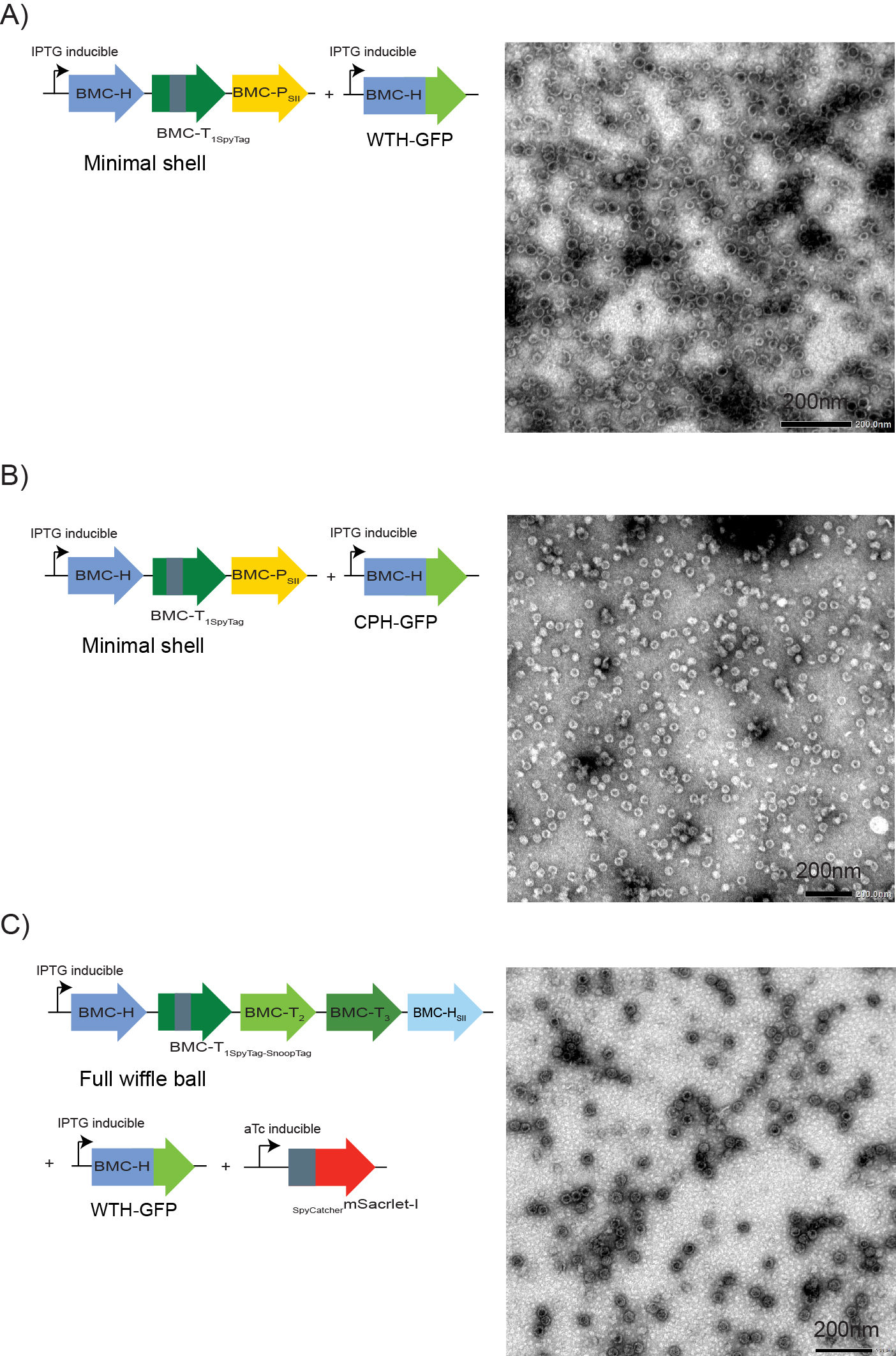

Supplement: Supplementary file 2 [file Image2.JPEG]
